# A Fine-Mapping Study of 7 Top Scoring Genes from a GWAS for Major Depressive Disorder

**DOI:** 10.1371/journal.pone.0037384

**Published:** 2012-05-23

**Authors:** Eva C. Verbeek, Ingrid M. C. Bakker, Marianna R. Bevova, Zoltán Bochdanovits, Patrizia Rizzu, David Sondervan, Gonneke Willemsen, Eco J. de Geus, Johannes H. Smit, Brenda W. Penninx, Dorret I. Boomsma, Witte J. G. Hoogendijk, Peter Heutink

**Affiliations:** 1 Department of Clinical Genetics, VU University Medical Center, Amsterdam, The Netherlands; 2 Department of Psychiatry, VU University Medical Center, Amsterdam, The Netherlands; 3 Department of Biological Psychology, VU University Medical Center, Amsterdam, The Netherlands; 4 Department of Psychiatry, Leiden University Medical Center, Leiden, The Netherlands; 5 Department of Psychiatry, University Medical Center Groningen, Groningen, The Netherlands; 6 Department of Psychiatry, Erasmus Medical Center, Rotterdam, The Netherlands; The Children's Hospital of Philadelphia, United States of America

## Abstract

Major depressive disorder (MDD) is a psychiatric disorder that is characterized -amongst others- by persistent depressed mood, loss of interest and pleasure and psychomotor retardation. Environmental circumstances have proven to influence the aetiology of the disease, but MDD also has an estimated 40% heritability, probably with a polygenic background. In 2009, a genome wide association study (GWAS) was performed on the Dutch GAIN-MDD cohort. A non-synonymous coding single nucleotide polymorphism (SNP) rs2522833 in the *PCLO* gene became only nominally significant after post-hoc analysis with an Australian cohort which used similar ascertainment. The absence of genome-wide significance may be caused by low SNP coverage of genes. To increase SNP coverage to 100% for common variants (m.a.f.>0.1, r^2^>0.8), we selected seven genes from the GAIN-MDD GWAS: *PCLO*, *GZMK*, *ANPEP*, *AFAP1L1*, *ST3GAL6*, *FGF14* and *PTK2B*. We genotyped 349 SNPs and obtained the lowest P-value for rs2715147 in *PCLO* at P = 6.8E−7. We imputed, filling in missing genotypes, after which rs2715147 and rs2715148 showed the lowest P-value at P = 1.2E−6. When we created a haplotype of these SNPs together with the non-synonymous coding SNP rs2522833, the P-value decreased to P = 9.9E−7 but was not genome wide significant. Although our study did not identify a more strongly associated variant, the results for *PCLO* suggest that the causal variant is in high LD with rs2715147, rs2715148 and rs2522833.

## Introduction

Major depressive disorder (MDD) is a psychiatric disorder characterized by persisting depressed mood, loss of interest or pleasure in normally enjoyable activities, psychomotor retardation and changes in e.g. sleep and appetite [1]. The lifetime prevalence in western civilization is estimated to be approximately 10–15% and the World Health Organization has predicted that by the year 2020, MDD will be the second leading cause of disability worldwide [2].

Though the etiology of the disease remains elusive, a genetic component is recognized and, based on twin studies, heritability is estimated to be around 40% [3]; [4]; [5]. However, MDD is a complex disorder and so far causal variants have proven to be difficult to find. For candidate genes, many association studies have been conducted, but this has not resulted in reproducible identification of susceptibility genes, because findings have often been inconsistent. This may be explained by methodological differences (i.e. difference in study design, study population, diagnostic criteria) or small sample sizes [6].

With the introduction of genome-wide association studies (GWAS), a systematic hypothesis-free search for common susceptibility genes became possible. The Netherlands Study for Depression and Anxiety and the Netherlands Twin Registry both took part in the Genetic Association and Information Network (GAIN) to conduct the first GWAS for MDD.

In this GWAS, 11 single nucleotide polymorphisms (SNPs) of the 200 SNPs with the lowest P-values located to a 167 kb segment overlapping the gene *PCLO*. This gene encodes the presynaptic protein piccolo, which has a possible role in facilitating monoamine transporter internalization [7]. In addition, it negatively regulates synaptic vesicle exocytosis by decreasing transport of vesicles from reserve pools to readily-releasable pools through an action on synapsin [8]. This suggests a possible role for *PCLO* in the regulation of mood-related monoaminergic neurotransmission.

Though multiple SNPs reached P-values in the order of 10E−7, genome-wide significance was not reached. 30 SNPs were included in a replication effort using an additional five MDD cohorts. These replication studies only partly confirmed the results. Only after post-hoc analysis with an Australian cohort that used similar ascertainment, the non-synonymous coding SNP rs2522833 showed nominal genome-wide significance (6.4E−8).

The lack of conclusive evidence for the involvement of any gene suggests that different factors are involved in different types of MDD. MDD is quite a heterogeneous disorder, with diagnosis based on levels of severity, depression subtypes and suggested underlying etiology. In order to obtain a more specific phenotype, one could use so-called endophenotypes: a concept with the purpose to divide for example behavioral symptoms into more stable phenotypes with a clearer genetic connection.

A second cause for sub-threshold P-values may be a lack of statistical power to detect a variant at a genome-wide level, due to the sheer number of variants genotyped. In addition, the effect size of a variant may be small in case of a common complex disorder. Thirdly, in order to accurately distinguish an association, it is imperative to have sufficient SNP-coverage within the regions of interest. Despite the intragenic association in *PCLO*, the SNP genotyping microarray that was used for the GWAS was not designed in a gene-centered manner. This implies that SNP coverage was generally not optimal for genic regions, including most genes for which small but not genome-wide significant p-values were found. We cannot rule out that these genes contain genetic risk factors, as there is no full coverage of them.

We therefore selected seven genes from the GAIN-MDD GWAS, with low SNP-coverage and multiple SNPs with a P-value ≤0.05, for further fine mapping. We aimed to increase coverage for these genes to capture all common variation in order to find a variant with stronger association with MDD in the GAIN-MDD cohort.

## Materials and Methods

### Samples

The subjects for this study originated from two longitudinal studies, the Netherlands Study for Depression and Anxiety (http://www.nesda.nl), designed to be representative of individuals with depression and/or anxiety disorders, and the Netherlands Twin Registry (http://www.tweelingenregister.org) for both of which sample collection and DNA isolation has been extensively described previously [9]; [10]. Genotyped samples contained 1738 cases and 1802 controls, of which 1216 male and 2324 female. All individuals had an age of 18–65 years and had self-reported western European ancestry.

### Ethical Issues

The NESDA and NTR studies were approved by the Central Ethics Committee on Research Involving Human Subjects of the VU University Medical Center, Amsterdam, an Institutional Review Board certified by the US Office of Human Research Protections (IRB number IRB-2991 under Federal-wide Assurance-3703; IRB/institute codes, NESDA 03–183; NTR 03–180). All subjects provided written informed consent. As part of the GAIN application process, consent forms were specifically re-reviewed for suitability for the deposit of de-identified phenotype and genotype data into the controlled-access dbGaP repository [11].

### Gene and Tag SNP Selection

We made a selection of the 25 genes with the lowest SNP P-values in the GAIN-MDD GWAS and ranked them according to 1) expression in the brain (yes or no), 2) high number of SNPs that reached P≤0.05 per total number of SNPs genotyped for this gene, 3) low SNP coverage of the gene in the GAIN-MDD GWAS, 4) low number of haplotype blocks per kb. Genes were tagged using the online Tagger tool [12] with r^2^>0.8 and m.a.f.>0.1 (Table 1). A margin of 5 kb around each gene was included, to tag possible regulatory regions as well. In addition, for each gene we included several SNPs that showed low P-values in the GAIN-MDD GWAS as a quality check.

**Table 1:** Selected genes, their function and the number of tag SNPs required to reach 100% coverage at m.a.f.>0.1 and r2>0.8.

Gene	Function/Description	Tag SNPs
*AFAP1L1*	Actin filament associated protein	21
*ANPEP*	Alanyl (membrane) aminopeptidase	17
*FGF14*	Fibroblast growth factor	167
*GZMK*	Granzyme K precursor	7
*PCLO*	Presynaptic active zone protein Piccolo	70
*PTK2B*	Protein tyrosine kinase	37
*ST3GAL6*	Beta-galactoside alpha-2,3-sialyltransferase 6	25

### Genotyping

Forty 96-well plates were made, blind to case-control status. Cases and controls were randomly allocated to plates and positions within plates. Each plate contained 93 samples from Dutch subjects, plus 3 QC samples at a concentration of 50 ng/µl of DNA. The three QC samples included two parents of one control sample on that plate, to add up to a total of 40 trios. Half of the plates contained a randomly selected duplicate case sample. Several samples were removed for analysis: offspring from trios, duplicates and various samples based on a principal component analysis described previously [10], leading to a total of 3540 samples (1738 cases and 1802 controls).

All genotyping was performed using the OpenArray® Real-Time PCR System (Life Technologies, Carlsbad, USA), in accordance with the protocol of the manufacturer (version: 7/2010). Arrays were designed to have 128 assays for 24 samples per array and were loaded using the OpenArray Accufill robot or using the AutoLoader, manually loaded into a cassette and then PCR was performed in an NT cycler (GeneAmp® PCR System 9700, Life Technologies, Carlsbad, USA). After this, arrays were scanned with the OpenArray NT Imager. 30 assays that were not correctly spotted onto the 128-format arrays were put on a separate 32-format array.

The quality of scanned arrays was checked by visually assessing the location of the array in the scanner (the so-called Spotfind image). The loading of the arrays was checked using the ROX image and the fluorescence signal strength was checked using the VIC and FAM images with the software tool ImageJ (http://rsbweb.nih.gov/ij/). Genotypes for approximately 200 samples were analyzed simultaneously, using Taqman Genotyper Software v 1.0.1. This number of 200 samples was set by optimizing for clear clustering, without getting a bias due to too few data points.

### Quality Control and Concordance Rates

For quality control reasons we included duplicated samples in the cohort. After genotyping we have checked the concordance between the two identical samples. To do this we used a home-made Perl script to compare all the genotype data from duplicate samples. Concordance was calculated for every SNP for which the sample and its duplicate both had a genotype. Concordance was 99.0% for duplicate samples. Out of the two duplicates we selected the sample that had the most genotype data for further analysis.

The Y-chromosomal SNP rs2534636 was included for QC. Genotype results for this SNP correspond to female/male distribution on the arrays.

Using the genome analysis tool PLINK, we performed quality control. As this is a follow up study of the initial GAIN-MDD GWAS, we chose to use the same quality control settings. Samples were excluded if more than 25% of data was missing, according to the standards that were used in the GAIN-MDD GWAS. SNPs were excluded if a) m.a.f. was lower than 1%, b) missing genotype rate was higher than 5%, c) more than one Mendelian error occurred in 38 trios, or d) P<10E−5 for the Hardy-Weinberg Equilibrium exact test in PLINK.

For each gene, several SNPs with a low P-value in the GAIN-MDD GWAS were also genotyped using the OpenArray system. Concordance between genotypes of both platforms was calculated to be 99.5% using PLINK [13].

### Statistical Analysis

The results of each analysis that was performed with the Taqman Genotyper Software were exported as a text file. Text files for all analyses were combined using a home-made script written in Perl [14]; With this script sample IDs, rs-numbers and genotypes were extracted and, with an additional script these data were merged into a ped-file.

All statistical analyses were performed using PLINK. We used an allelic chi-square test with one degree of freedom to perform association analysis, to compare the allele frequencies between MDD cases and controls for each SNP. Since this project entails the fine mapping of the results of a GWAS, we corrected for genome-wide significance when performing the association analysis. A P-value of 5E−8 or lower was considered to be genome-wide significant.

Haplotype blocks were calculated with PLINK, using the method of Gabriel et al., which defines pairs to be in strong LD if the one-sided upper 95% confidence bound on D′ is larger than 0.98 and the lower bound is above 0.7 [15]. The association of haplotypes with MDD was calculated with a chi-square test using one degree of freedom.

### Calculation of Coverage

In order to calculate coverage, we used the online Tagger tool from De Bakker et al [12]. We force included all the tag SNPs that we selected and force excluded all other SNPs for tagging at m.a.f.>0.1 and r^2^>0.8. This resulted in a calculation of how many SNPs out of all the present SNPs are covered by the force included tag SNPs.

### Imputation

MaCH was our imputation method of choice, based on its high imputation accuracy and efficacy, its user-friendly data handling [16], and high compatibility with 1000 genomes data. 1000 genomes 2010-06 release CEU data was used as a reference, because of its high number of variants and its novelty [17].

We did not use imputation data for the entire chromosome, as we were only interested in seven genes and their regulatory regions. However, to leave the underlying LD-structure intact, we used a margin of 100 kb around each gene.

To extract the genes +/−100 kb from the full chromosome data of the 1000 genomes project, we used a home-made script written in Python [18]. According to MaCH protocol, an estimation of imputation parameters was created with 100 random control samples and 100 random cases, to get information about the length of haplotype stretches shared between our data and the reference panel [19]. After estimating parameters, imputation was performed with 100 Markov chain iterations for the entire cohort, per gene. All imputation was performed on the Lisa system cluster (www.sara.nl/systems/lisa). For each gene, we filled in the missing genotypes by imputation and left genotyped SNPs intact.

### Joint Reanalysis

We performed a joint reanalysis of 77 *PCLO* SNPs surrounding rs2522833 and rs2715147. For this analysis, we calculated Z-scores by performing logistic regression and dividing the slope for each data point by its standard error, similar to the method used by Sullivan et al [10]; [20]. The absolute values of these Z-scores were then plotted against the root of the r^2^ between one of these 77 SNPs with either rs2522833 or rs2715147.

### Epistasis Analysis

To perform an analysis of epistasis, we selected 52 genes that, on a protein level, interact with *PCLO*, using the method of Lips et al. [21] for 47 synaptic genes and using the InWeb database [22] for 5 additional genes. Genotypes for the SNPs existing in these genes were extracted from the GAIN-MDD GWAS data, after which epistasis analysis was performed with PLINK [13]. We tested a total of 94 *PCLO* SNPs against the 1579 SNPs in the selected genes.

## Results

### Genotyping

The seven selected genes were tagged in order to reach 100% coverage at r^2^>0.8 and m.a.f.>0.1. A total of 349 tag SNPs were selected for genotyping. After genotyping, five SNPs were removed due to poor clustering. 51 SNPs and 64 samples failed because of high levels of missing data, after which the average call rate per sample was 96.7% and average call rate per SNP was 96.7%.

### Coverage

In order to compare the coverage of the seven selected genes, coverage was calculated before and after additional genotyping, using the online Tagger tool [12]. SNPs that were genotyped in the original GWAS were merged with the 298 SNPs that were genotyped and passed the quality control that we performed with the genome analysis tool PLINK [13]. After merging, 459 SNPs and 3476 individuals remained (1712 cases and 1764 controls) for the seven genes. Total genotyping rate in remaining individuals was 98.8%.

As not all our tag SNPs passed quality control, we did not reach 100% coverage for all genes, but adding these SNPs to those genotyped in the initial GWAS resulted in significantly higher coverage (Table 2).

**Table 2:** Coverage calculated for each gene at r^2^>0.8 m.a.f.>0.1 before and after fine mapping.

Gene	Coverage after GAIN-MDD GWAS	Coverage with additional genotyping
*AFAP1L1*	50%	75%
*ANPEP*	68%	93%
*FGF14*	50%	94%
*GZMK*	80%	100%
*PCLO*	88%	95%
*PTK2B*	83%	98%
*ST3GAL6*	80%	93%

### Association Analysis

After quality control, we performed association analysis for the newly genotyped SNPs using PLINK, for association with MDD. For six genes the result of fine mapping did not improve P-values compared to the P-values that were detected in the original GAIN-MDD GWAS (Table 2). However, for *PCLO* we found that rs2715147 had a P-value of 6.8E−7. This is lower than rs2715148, which showed the lowest P-value (P = 7.7E−7) for *PCLO* in the GAIN-MDD GWAS. This finding did not reach genome-wide significance (P = 5E−8).

We then compared rs2715147 and rs2715148, while only using the samples that were genotyped for both SNPs to exclude a bias due to unequal numbers of cases and controls. We thus excluded all samples with missing genotypes for either of these SNPs, after which rs2715148 had a P-value of 5.3E−7 and rs2715147 had a P-value of 6.8E−7.

As 51 SNPs were excluded from the analysis after quality control, this prevented reaching full coverage for 6 genes, except for *GZMK*. To increase coverage for these genes after exclusion of these SNPs, we imputed missing genotypes using the 1000 genomes CEU data.

After imputation we again performed an association analysis (Table 3). rs2715147 and rs2715148 showed a similar P-value: 1.223E−6. In addition, the P-values for *FGF14* and *PTK2B* decreased. However, none of the genotyped and imputed SNPs reached genome-wide significance (P = 5E−8). After imputation, rs2715147 and rs2715148 show a slightly better P-value (P = 1.172E−6) than the non-synonymous coding SNP rs2522833 (P = 1.223E−6). When using a logistic model with sex as a covariate, P-values for rs2715147 and rs2715148 increased slightly to P = 1.763E−6, showing only a marginal effect of sex when taken along as a covariate.

**Table 3:** rs-numbers and P-values for the SNPs with the lowest P-values.

Gene	GAIN-MDD^a^	P-value	Fine mapping^b^	P-value	OR; CI	Imputed data^c^	P-value	OR; CI
*AFAP1L1*	rs4705335	1.9E−4	rs352355	1.3E−2	0.83; 0.72–0.96	rs4705335	2.7E−4	1.26; 1.11–1.43
*ANPEP*	rs6496603	5.6E−5	rs8035089	3.9E−4	0.82; 0.72–0.92	rs6496603	5.7E−5	0.82; 0.75–0.90
*FGF14*	rs17688345	1.2E−4	rs9518638	1.6E−3	0.84; 0.75–0.94	rs17688345	8.2E−5	0.75; 0.65–0.87
*GZMK*	rs2112938	5.1E−5	rs6875666	4.9E−3	0.86; 0.78–0.96	-	-	-
*PCLO*	rs2715148	7.7E−7	rs2715147	6.8E−7	0.79; 0.72–0.87	rs2715147+ rs2715148	1.2E−6	0.79; 0.72–0.87
*PTK2B*	rs7000615	1.5E−4	rs748281	3.7E−4	1.30; 1.12–1.50	rs7000615	5.4E−5	1.30; 1.14–1.47
*ST3GAL6*	rs999147	1.6E−4	rs704586	1.0E−3	0.84; 0.76–0.93	rs14310	1.7E−4	1.2; 1.09–1.33

a GAIN-MDD GWAS, ^b^tag SNPs used for fine mapping, ^c^both GAIN-MDD GWAS and fine-mapping tag SNPs, merged and imputed. OR =  Odds Ratio, CI =  Upper and Lower bounds of the 95% Confidence Interval.

### Haplotypes

Using PLINK, we calculated the architecture of haplotype blocks for each gene, for the genotype data completed with imputed data (Table 4). With this data, again an association test was performed. This showed a decrease in the P-values for *AFAP1L1* and *FGF14*, but did not reach genome-wide significance for any of the genes.

**Table 4:** The haplotypes with the lowest P-values, per gene.

Gene	SNPs in haplotype block with lowest P-value	P-value
*AFAP1L1*	rs10515625|rs4705335|rs12657199|rs1438693|rs11954165|rs1438692	1.7E−4
*ANPEP*	rs8035089|rs10584|rs6496603|rs17239917|rs25651|rs16943599|rs1439120	2.8E−4
*FGF14*	rs17688345|rs9518615|rs9557792|rs636674|rs1457315|rs4772439|rs35700852|rs7992504|rs12865694	2.5E−5
*GZMK*	rs3776038|rs6875661|rs6875666|rs2112938	9.9E−5
*PCLO*	rs2715147|rs2715148|rs2522833|rs2522840|rs2522843|rs7792042|rs12707523|rs12707524|rs13233504	2.0E−6
*PTK2B*	rs7827965|rs9773817|rs3736524|rs11135993	7.0E−4
*ST3GAL6*	rs3821359|rs2334230|rs278376|rs3755574|rs16846347|rs3755576|rs999147|rs828609|rs278390|rs14310|rs704586	2.4E−4

### Joint Reanalysis

**Figure 1:**
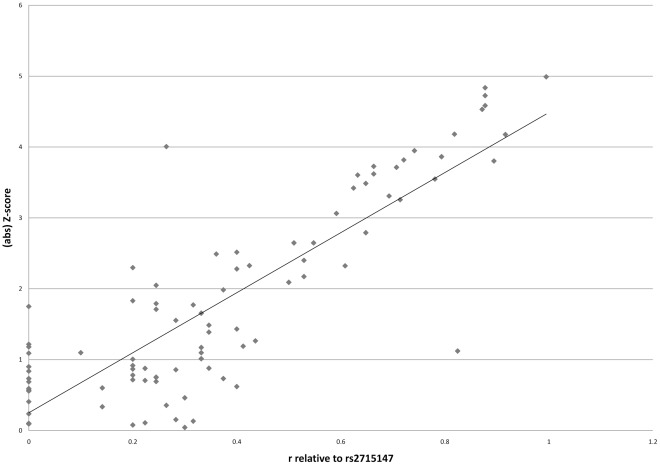
Linear fit for the Z-scores and correlation (√r^2^) between markers and rs2715147. The linear fit with Z-scores versus r relative to rs2715147, for 77 markers in *PCLO*.

In addition, we performed a joint reanalysis of 77 SNPs surrounding rs2522833 and rs2715147. The absolute values of Z-scores were plotted against the square root of the r^2^ between one of these 77 SNPs with either rs2522833 or rs2715147. When assuming the null-hypothesis of no association, one would expect that the slope of the linear fit would approximate 0, since SNPs in high LD with a causal variant will reflect the Z-score of this causal variant. When we assume that rs2522833 is the causal variant, the slope of the linear fit is 4.17, which increases slightly to 4.24 when assuming that rs2715147 is the causal variant (Figure 1), supporting the hypothesis that an unknown variant between rs2715147 and rs2522833 may be causal for MDD in the GAIN-MDD cohort.

### Epistasis Analysis

Since *PCLO* gave the lowest P-values of the seven genes selected for fine mapping, epistasis analysis was performed for *PCLO* only. The lowest P-value (1.6E−05; OR 0.5928) was found for *PCLO* SNP rs6947662 in conjunction with rs16946196, which is located in *DLGAP1*. Since this epistasis analysis did not lead to a lower P-value than a single SNP analysis, we found no evidence for an epistatic effect of *PCLO* SNPs with SNPS from interacting proteins.

**Figure 2:**
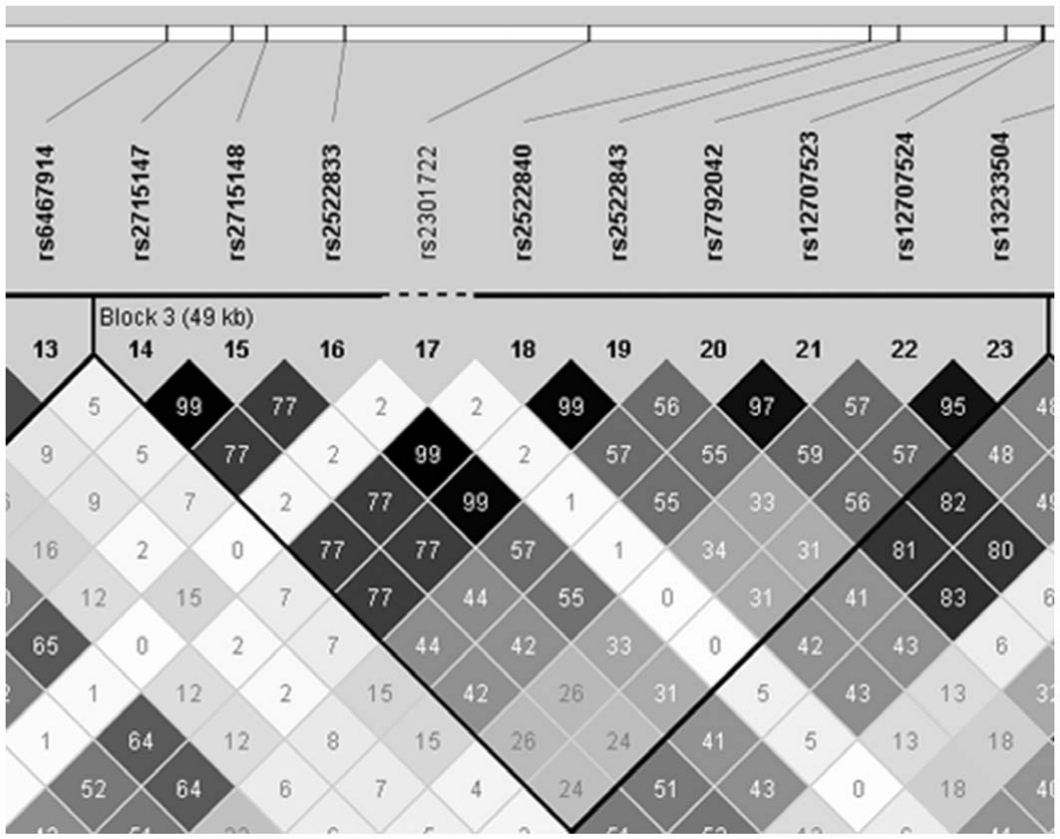
The LD-structure of *PCLO*. The LD-structure of *PCLO* shown in an r^2^-plot created in Haploview. The plot shows the LD-block in which the SNPs with the lowest P-values were found. Non-synonymous coding SNP rs2522833, rs2715147 and rs2715148 are in high r^2^ with each other.

Using the merged data of the GAIN-MDD GWAS, our fine mapping study, plus the imputed data, we generated an r^2^-plot of the region spanning *PCLO* in the haplotype analysis program Haploview [23], since *PCLO* provided the lowest P-value. rs2715147 and rs2715148 are in high r^2^ (0.99) with one another. In addition, both SNPs show an r^2^ of 0.77 with the non-synonymous coding SNP rs2522833 (Figure 2).

Based on the haplotype structure as seen in Haploview, we performed a haplotype association test with for rs2715147, rs2715148 and rs2522833, as we find the lowest P-values in this region. For this haplotype we found a P-value of 9.9E−7, meaning that the combination of these three SNPs as a haplotype will give a slightly better association than any of them as a single SNP.

## **Discussion**

In 2009 a GWAS for MDD was performed [10]. Unfortunately, the propriety microarrays used for this GWAS (Perlegen Sciences Inc., Mountain View, CA, USA) where not designed in a gene-centered manner resulting in incomplete coverage of genic regions. From the 25 genes that harbored the SNPs with lowest P-values, we selected seven genes for fine mapping. We used the Hapmap Tagger tool to tag these genes with r^2^>0.8 and m.a.f.>0.10, in order to capture all common variation.

After genotyping, several SNPs were excluded through quality control. Even though we did improve coverage significantly, due to this exclusion we did not acquire full coverage for all genes. Full coverage was reached only for *GZMK*. We performed an association test with all SNPs and samples that made it through cut-off values. For the SNP rs2715147 in *PCLO* we found a P-value of P = 6.8E−7, which is lower than the lowest P-value for *PCLO*-SNPs in the original GAIN-MDD GWAS (P = 7.7E−7 for rs2715148). This small decrease in P-value could also be due to technical variability, however, in both the GAIN-MDD GWAS and our fine mapping project, the lowest P-values are found in this area of the *PCLO* gene. For the other six genes we did not find a variant with better association than in the GAIN-MDD GWAS.

Since we reached 100% coverage only for the *GZMK* gene, we filled up missing genotypes by performing imputation with MaCH for the remaining six genes. Previously, for the GAIN-MDD GWAS, two imputation approaches have been used: MaCH was used for imputing 2037829 autosomal SNPs with r^2^≥0.5 (which removes approximately 90% of SNPs with unreliable imputation results, while dropping only 2–3% of reliably imputed SNPs) and using the SNPMStat method [24], 246 SNPs in the *PCLO* area were imputed. The HapMap2 CEU panel was used as a reference [10].

In this study, imputation was only performed for missing genotypes, rather than for all new tag SNPs. The rationale behind this is that there are local differences in LD structure between the GAIN-MDD cohort and the HapMap CEU population. This might decrease the validity of the genotypes estimated by imputation [25].

We used MaCH to impute for six genes. Imputation decreased P-values for *FGF14* and *PTK2B*, albeit for the SNP that showed the lowest P-value for those genes in the original GAIN-MDD GWAS. For *PCLO*, rs2715147 and rs2715148, which are in strong LD, both showed the same P-value at P = 1.2E−6, which was also the lowest P-value for this gene. For *ANPEP*, *AFAP1L1* and *ST3GAL6*, P-values were not improved by means of imputation. None of the genes showed a genome-wide significant association with MDD after imputation.

In addition, we wanted to investigate whether SNPs in *PCLO* are interacting with SNPs in synaptic genes. To determine this, we performed an epistasis analysis using PLINK [13]. As the lowest P-value was in the range of 10E−5, we cannot conclude whether there is epistasis between these SNPs or not.

In a joint reanalysis of 77 *PCLO* SNPs we show a graphical representation of the Z-scores for each SNP versus the correlation of this SNP with rs2715147. In comparison with rs2522833, the slope for rs2715147 is slightly steeper. This supports the hypothesis that the low P-values in this area may be caused by an unknown variant located between rs2715147 and rs2522833, or an unknown variant that is in strong LD with these SNPs.

We can conclude that fine mapping of these seven genes did not provide a variant with a stronger association than reported in the original GAIN-MDD GWAS, where the lowest P-value was obtained for rs2715148 and rs2522833 showing nominal significance after post-hoc analysis with an Australian cohort. However, there could be a number of reasons for this apparent lack of association. First of all, diagnosis of MDD is based on relatively subjective assessments of symptoms. By specifying endophenotypes within an MDD cohort, for instance brain activity, cortisol levels and pharmacological response, one might find variants that are exclusive to that particular endophenotype, with a higher effect size.

Another possibility would be to expand the cohort in order to increase the power for detecting an associated variant. Park et al. show that for a number of complex traits, the sample size has to be at least around 10,000 in order to reliably detect new variants [26].

One way to create such an expansion would be to perform a meta-analysis of several cohorts. Nevertheless, despite the increase in sample size, one has to take into account that a meta-analysis, in case of MDD, may also increase any heterogeneity caused by inconsistencies in ascertainment.

Other GWAS for depression are troubled by equal predicaments. So far, marginally significant associations have been found for -among others- *FKBP5*, *SP4*, *GRM7*, *C5ORF20* and *NPY.* However, many of these results cannot be replicated in another cohort [27]; [28]; [29]; [30]. Here again, sample size may be crucial to acquire the statistical power necessary to find an associated variant. In addition, not all studies use the same method of ascertainment. Even though cases are mostly obtained for research through a DSM-IV diagnosis of MDD, more specific secondary interviews may deviate in determining depression subtypes, severity, age of onset, recurrence and comorbidity [28].

Although we did not select our seven genes based on their function, several of them are linked to the central nervous system and brain physiology. First of all, the product of *ANPEP*, aminopeptidase N, metabolizes angiotensin III (AngIII), which is one of the main effector peptides of the brain renin-angiotensin system. This system controls vasopressin release in the brain. When aminopeptidase N is inhibited, both AngIII and vasopressin increase, which in turn causes an increase of ACTH [31]. An increase in ACTH ultimately stimulates the release of cortisol, which is a major stress hormone. This connects aminopeptidase N to the HPA-axis, which is linked to MDD as it elicits the stress-response in the brain [32].

Both *PTK2B* and *GZMK* have been linked to brain physiology and depression through animal models. Following acute stress, *PTK2B* (also known as pyk2) expression is increased, whereas increasing *PTK2B* activity in lateral septum neurons reverses the behavioral deficits of acute, inescapable stress. These findings establish a role for *PTK2B* in the behavioral response to stress and may suggest a possible role in the pathophysiology of depression [33].

*GZMK* is part of a network of genes that are co-expressed higher in mice that have a high predisposition to freezing behavior or catalepsy [34]. This reaction is a natural passive defensive strategy, but in chronically stressed animals, for instance in models for post-traumatic stress disorder or MDD, animals show enhanced catalepsy [35].

The protein product of *PCLO*, Piccolo, can be found in the presynaptic active zone [36]. If Piccolo is knocked out, synapse formation or morphology is not affected, suggesting that piccolo is not necessary for formation of synapses. However, synapses lacking Piccolo exhibit faster rates of synaptic vesicle exocytosis, indicating that Piccolo is a negative regulator of the exocytotic process [8]. This may suggest a role for Piccolo in the monoamine hypothesis of depression, which states that depression is caused by an imbalance of monoamine availability [37]. In addition, the non-synonymous coding SNP that was found to be significant in the GWAS by Sullivan et al. [10], changes a serine to an alanine in a calcium-binding C2A-domain. Overexpression of this C2A-domain causes a depression-like phenotype in mice [37].

These genes may still be interesting candidate genes, when looking at monoamine availability (*PCLO*), or more specific (endo-)phenotypes like cortisol levels (*ANPEP*) and co-morbid anxiety (*PTK2B* and *GZMK*). Despite the fact that the other selected genes that are also expressed in the brain, based on exploring literature, they do not show an obvious link with MDD. In combination with their apparent lack of genome-wide associated variants, this makes them less likely to be successful candidate genes.

None of the SNPs for any of the seven genes showed a P-value in the magnitude of P = 5E−8, which leads to the conclusion that in the scenario of common variation and corrected for genome-wide testing, these genes show no genome-wide significant association with MDD for this cohort. However, considering the fact that in *PCLO* there are several signals in the magnitude of P = 1.0E−6 and P = 1.0E−7 and the “Fundamental Theorem of the HapMap”, which states that all tested SNPs are expected to reflect the true association of the unknown causal variant proportional to their LD with it, one cannot disregard the possibility that a rare variant may still be associated.

Previously, we showed that most of the association between genotype data and MDD is statistically explained by the association of the non-synonymous coding SNP rs2522833 with MDD. The data from the GWAS are consistent with the hypothesis that either rs2522833 or a variant in high LD with it is a causal risk factor for MDD [38]. However, our data do not favor rs2522833 as the causal variant, as it does not show the lowest P-value in our data set. We do see a very high LD (r^2^ = 0.99) between rs2715147 and rs2715148 and a high LD between these two SNPs and rs2522833 (r^2^ = 0.77). In addition, the haplotype which includes SNPs rs2715147, rs2715148 and rs2522833 shows a lower P-value than the P-values calculated for these SNPs individually. This implies that between rs2715148 and rs2522833 there may be an unknown variant that has an r^2^ of at least 0.77 with both variants and has a slightly better association with MDD (9.9E−7). Nevertheless, this observation could also be caused by missing data. Ideally, the study should be replicated in a larger cohort or in a meta-analysis in order to confirm or decline the improved P-value in case of this haplotype.

In addition, instead of looking at SNPs as individual units of association studies, one might jointly analyse all variants within a putative gene to obtain a single P-value for the association of the entire gene, as it is the functional unit of the genome. A pitfall for joint analysis is that one would have to assign weights to the individual SNPs, as not every SNP will have the same impact on a putative association. In tools for gene-based P-values, this matter is still an open question, as we do not yet have a full understanding of the relationship between sequence and function [39].

In conclusion, the current study suggests that using common variation to fine map the GAIN-MDD GWAS results, does not lead to lower P-values or the identification of a stronger associated variant. The genomic region in *PCLO* between rs2715147 and rs2522833 covers approximately 5 kb. It is estimated that SNPs occur every 100–300 bp in the human genome. That would imply that between rs2715147 and rs2522833 approximately 16–50 variants could occur. With new, powerful approaches for DNA analysis such as next generation or massive parallel sequencing (MPS), these variants could be identified and subsequently genotyped in the whole cohort. This could lead to the discovery of a causal variant that is in high LD with rs2715147, rs2715148 and/or rs2522833. Accordingly, we should perform MPS for *PCLO*, in order to confirm the existence of such a variant and find its association with MDD.
